# From policy to practice: syndemic and intersectional challenges to ART adherence for transgender women under India’s post-test and treat policy

**DOI:** 10.1080/17441692.2025.2473446

**Published:** 2025-03-06

**Authors:** William Lodge, Jatin Chaudary, Shruta Rawat, Madina Agénor, Alpana Dange, Vivek R. Anand, Don Operario, Matthew J. Mimiaga, Katie B. Biello

**Affiliations:** a Jeb E. Brooks School of Public Policy, Cornell University, Ithaca, NY, USA; b Cornell Center for Health Policy, Cornell University, Ithaca, NY, USA; c The Humsafar Trust, Mumbai, Maharashtra, India; d Department of Behavioral and Social Health Sciences, Brown University School of Public Health, Providence, RI, USA; e Center for Health Promotion and Health Equity, Brown University School of Public Health, Providence, RI, USA; f The Fenway Institute, Fenway Health, Boston, MA, USA; g Department of Behavioral, Social, Health Education Sciences, Emory University Rollins School of Public Health, Atlanta, GA, USA; h Department of Epidemiology, UCLA Fielding School of Public Health, Los Angeles, CA, USA; i UCLA Center for LGBTQ+ Advocacy, Research & Health, Los Angeles, CA, USA; j Department of Epidemiology, Brown University School of Public Health, Providence, RI, USA

**Keywords:** People living with HIV, India, syndemics, intersectionality, transgender women, SDG 3, good health and well-being, SDG 10, reduced inequalities, SDG 5, gender equality

## Abstract

Transgender women (TGW) in India face one of the highest HIV prevalence rates among key populations in India, yet data on their engagement in the HIV care cascade is limited. This study investigates barriers and supportive factors for adhering to antiretroviral therapy (ART), which is vital for achieving viral suppression, reducing transmission risk to nearly zero (i.e. undetectable = utransmittable; U = U), and enhancing the quality of life for TGW living with HIV. Between July and September 2023, trained community recruiters recruited 30 TGW living with HIV in Mumbai and New Delhi, India. Using intersectionality and syndemic theory as guiding frameworks, we purposively sampled and conducted semi-structured qualitative interviews. The interviews revealed four main themes – two barriers and two supportive factors influencing ART adherence: the impact of poverty on syndemic factors, intersectional stigma and discrimination, empowerment to overcome barriers, and the influence of inclusive government programmes and policies in improving TGW’s access to ART. Despite the availability of free ART immediately after diagnosis under India’s ‘test and treat’ policy, economic instability and intersecting stigma hinder adherence. Our findings reveal that holistic interventions focusing on economic support, stigma reduction, and personal and collective empowerment might improve ART adherence among TGW in India.

## Introduction

Globally, transgender women (TGW) experience a high burden of HIV compared to their cisgender counterparts (Poteat et al., 2016). This disparity is also evident in India, which has the third-largest HIV epidemic in the world. The prevalence of HIV among TGW in India is 4%, far exceeding the national average of 0.2% (National AIDS Control Organization, 2023). In this paper, we use the term ‘transgender women’ broadly to include other culturally and historically significant gender non-conforming identities in India, such as hijras, aravani, and jogti, which are deeply rooted in South Asia’s rich cultural, historical, and religious traditions.

India has a robust antiretroviral therapy (ART) programme that provides free ART to all people living with HIV through government ART centres (National AIDS Control Organization, 2023). These ART centres dispensed ART monthly. However, individuals who demonstrated optimal adherence (i.e. regularly taking ART as prescribed) and maintained a suppressed viral load, are qualified for multi-month dispensing, which can provide 2–3 months of ART at a time (National AIDS Control Organization, 2023). The 2017 implementation of the ‘test and treat’ policy, providing ART immediately after diagnosis, transformed HIV care linkage in India (National AIDS Control Organization, 2023; National AIDS Control Organization & ICMR-National Institute of Medical Statistics, 2018). A recent cost-effectiveness analysis study of heterosexual individuals living with HIV showed that the implementation of the policy averted 18,386 HIV-related deaths and 16,105 new HIV infections and saved 343,172 quality-adjusted life years (QALYs) compared to previous years when the ART initiation cut-off was 500/mm^3^ (Singh et al., 2022).

However, despite these advancements, India remains far below the UNAIDS 2030 95–95-95 targets, with only 77% of individuals aware of their HIV status, 65% on treatment, and 55% achieving viral suppression (UNAIDS, 2021). Importantly, a critical gap exists in understanding the HIV care cascade among TGW living with HIV in India (Chakraborty et al., 2020). This gap is further illustrated by ART coverage estimates, which indicate approximately 70% coverage in the general population but only 58% among transgender people (UNAIDS, 2024).

While the ‘test and treat’ policy was designed to improve access, persistent barriers remain. Qualitative studies have explored these barriers and facilitators, focusing primarily on other key populations, such as female sex workers, men who have sex with men, and people who use drugs (Acharya et al., 2024; Joglekar et al., 2011; Kumarasamy et al., 2005). However, studies exclusively examining TGW are limited (Acharya et al., 2024; Azhar et al., 2024; Chakrapani et al., 2023). This highlights the need for research to understand barriers and facilitators specific to TGW. Using qualitative interviews, this study seeks to address the existing gap in knowledge by conducting an in-depth exploration of the barriers and supportive factors to ART adherence among TGW living with HIV in India.

The challenges TGW face are particularly pronounced in urban environments, where structural and social vulnerabilities intersect. Studies have shown that higher HIV prevalence is often associated with greater urbanisation (Joshi & Mehendale, 2019; Kumar et al., 2017; Perkins et al., 2009). This association stems from factors such as separation from families, which disrupts social support networks, increased involvement in sex work as a means of economic survival, and a lack of accessible information about HIV prevention (Joshi & Mehendale, 2019; Ranjan et al., 2017). These dynamics are particularly pronounced among key populations, including TGW, who are disproportionately affected by structural and social vulnerabilities in urban environments(Chakrapani, 2010).

Few studies examine the association between these factors on ART adherence among TGW in India, and fewer studies reflect the post-2017 ‘test and treat’ policy (Chakrapani et al., 2011, 2017, 2023; Ganju & Saggurti, 2017; Piña et al., 2018; Shaw et al., 2016; Willie et al., 2017). Barriers such as occupation, conformity to gender norms, caste, and skin colour are linked to community health outcomes and contribute to ART adherence disparities (Johnson, 2002; Uddin et al., 2020). However, studies exploring these intersecting factors and their impact on ART adherence remain sparse.

To address these complex challenges, this study employs intersectionality and syndemic theory to understand barriers to ART adherence. Intersectionality provides a nuanced examination of the compounding effects of multiple co-constituted systems of oppression and power relations on the lived experience of multiple marginalised groups (Bowleg, 2021; Crenshaw, 1991). Moreover, syndemic theory posits how two or more psychosocial and contextual factors synergistically affecting health outcomes (Singer & Clair, 2003). These frameworks provide complementary perspectives, illustrating the complex dynamic interplay that elevates the role of socio-structural determinants and group-level complexity contributing to population-based inequities in HIV and, more specifically, ART adherence (Smith et al., 2022).

Additionally, this study aims to examine supportive factors that enable TGW living with HIV to overcome barriers to ART adherence. By focusing on personal and collective empowerment, this study highlights how TGW can navigate structural challenges and improve access to resources. These insights aim to inform tailored interventions and policies to advance equitable HIV care for TGW in India.

## Methods

### Setting and participants

From July to September 2023, we conducted 30 in-person, in-depth interviews with TGW living with HIV in Mumbai and New Delhi, India. These cities have HIV prevalence among TGW reaching 12% and 5%, respectively, significantly higher than in other cities in India (National AIDS Control Organization, 2016). The inclusion criteria were as follows: (1) 18 years or older; (2) living with HIV for at least 1 year; (3) assigned male sex at birth; (4) currently identifies as a woman, transgender woman, hijra, or another identity on the trans-feminine spectrum; (5) ever prescribed ART; (6) able to provide informed consent both written and audio recorded; and (7) speaks English, Hindi, and/or Marathi. Purposive sampling was to recruit participants with diverse perspectives and experiences based on intersecting identities within the larger TGW community (e.g. economic status, occupation, indigenous gender-diverse identities such as hijras, and religion) (Patton, 2015). Transmasculine and nonbinary people were excluded from the study, as their distinct socio-cultural and epidemiological contexts warrant separate studies to adequately address their unique experiences and barriers.

Participants were recruited by trained peer outreach recruiters at The Humsafar Trust (HST), one of India’s largest LGBTQ + community-based organizations (CBO), providing clinical and social services to over 4000 TGW annually in Mumbai and New Delhi. We utilised different recruitment strategies in each city: in Mumbai, we recruited through community networks and health interventions based at HST, while in New Delhi, we employed community networks and liaised with other local CBOs who provided social welfare services to TGW living with HIV in New Delhi.

### Reflexivity statement

This study was conceptualised and conducted in close collaboration with HST, ensuring that the research was grounded in the lived realities and priorities of TGW living with HIV. From its inception, our community partners played a pivotal role in shaping the research design, including selecting New Delhi as a site to address the lack of data on TGW living with HIV in this city. The US-Indo research team prioritised reflexive practices, recognising our positionalities and the potential for power dynamics to influence data collection. To mitigate this, we intentionally hired research staff and interviewers from the LGBTQ + community, ensuring that TGW were involved in recruiting participants and conducting the interviews. These efforts ensured that the study was both methodologically rigorous and community-centered, exemplifying a collaborative approach.

### Data collection

We developed an interview guide based on a conceptual model grounded in syndemic theory and intersectionality, focusing on barriers and supportive factors influencing HIV care outcomes (Lodge II et al., 2025). The guide included open-ended questions and probes on topics such as general health, experience receiving HIV care, and barriers (e.g. intersectional stigma, psychosocial problems, unmet gender-affirming care) to ART adherence. The interview guide was pre-tested in Mumbai and New Delhi with HST staff who provided lived experience and local expertise.

At the start of the study visit, all participants provided informed consent. Only the interviewer and participants were present during the visits conducted in a private space at HST. Before the interview, we collected a brief demographic and self-reported ART adherence survey to contextualize the qualitative data. Interviews were conducted in local languages (i.e. Hindi and Marathi) by trained research assistants at HST and ranged from 45 to 60 min in duration at HST. The research assistants were local experts with experience conducting qualitative interviews, with at least 50% identifying as a part of the LGBTQ + community. Interviews were audio-recorded using a handheld audio recorder. A professional transcription company transcribed the audio recordings verbatim and translated them into English. Participants received INR 500 (∼7 USD) for their time and provided refreshments at the interview. Research assistants and the project manager, fluent in Hindi and Marathi, quality-checked the interviews to ensure the accuracy of the translation and transcription process. A research assistant knowledgeable in Hijra Farsi, a language exclusively spoken among hijras, also quality-checked transcriptions to ensure the translation was accurate. The human subjects research review boards reviewed and approved the study at HST and Brown University.

### Data analysis

The analysis of interview transcripts followed a template-style thematic analysis approach, which integrated both deductive and inductive coding approaches (Crabtree & Miller, 1992; King, 2004), utilising a hierarchical codebook derived from the conceptual framework (Lodge II et al., In press). The data analysis started with data immersion and developing, testing, and refining the codebook using a subset of the interviews. All efforts in crafting the codebook were completed in collaboration with HST (Appendix I). To ensure the reliability of our coding process, two independent coders initially applied the codebook to five transcripts as a test to see if there were any discrepancies. Any discrepancies were discussed and resolved through consensus. Following these discussions, the codebook underwent further refinement and was applied consistently to the remaining data (Crabtree & Miller, 1992; King, 2004). The coded text fragments, specifically those addressing barriers and supportive factors to ART adherence, were systematically organised into overarching themes and sub-themes, which will be used throughout the manuscript (Fereday & Muir-Cochrane, 2006). All themes and sub-themes were discussed with the research team and revised based on these discussions.

## Results

### Participant sociodemographic characteristics and self-reported ART adherence

Table 1 presents diverse participant ages, with the majority falling between 25 and 44 years old (64%). Most participants identified as TGW (63%), including a significant subgroup of hijra individuals (37%), a culturally distinct identity within the transgender community. The majority held secondary school (Year 10) and higher secondary certificates (Year 12) (60%). While 30% reported being perceived as conforming to their affirmed gender, 27% stated that they rarely felt they were perceived this way. Most participants said their perceived skin colour was ‘Medium/Dusky’ (57%), followed by ‘Fair’ (23%). Monthly incomes ranged from ‘less than 5,000 INR’ (10%) to ‘more than 20,000 INR’ (7%). All participants reported a lifetime history of engagement in sex work, and 73% reported engagement in sex work in the past 3 months.

While all participants reported being currently on ART, there were disparities in initiation and adherence patterns. Despite the ‘test and treat’ policy’s goal of promoting immediate ART initiation, self-reported ART adherence varied in the past month, with 37% reporting ‘Good’ and ‘Excellent’ compliance. Most participants (80%) reported ART adherence above 90%, while 40% reported perfect adherence (100%) (Table 1).

### Research findings

**Figure 1.**
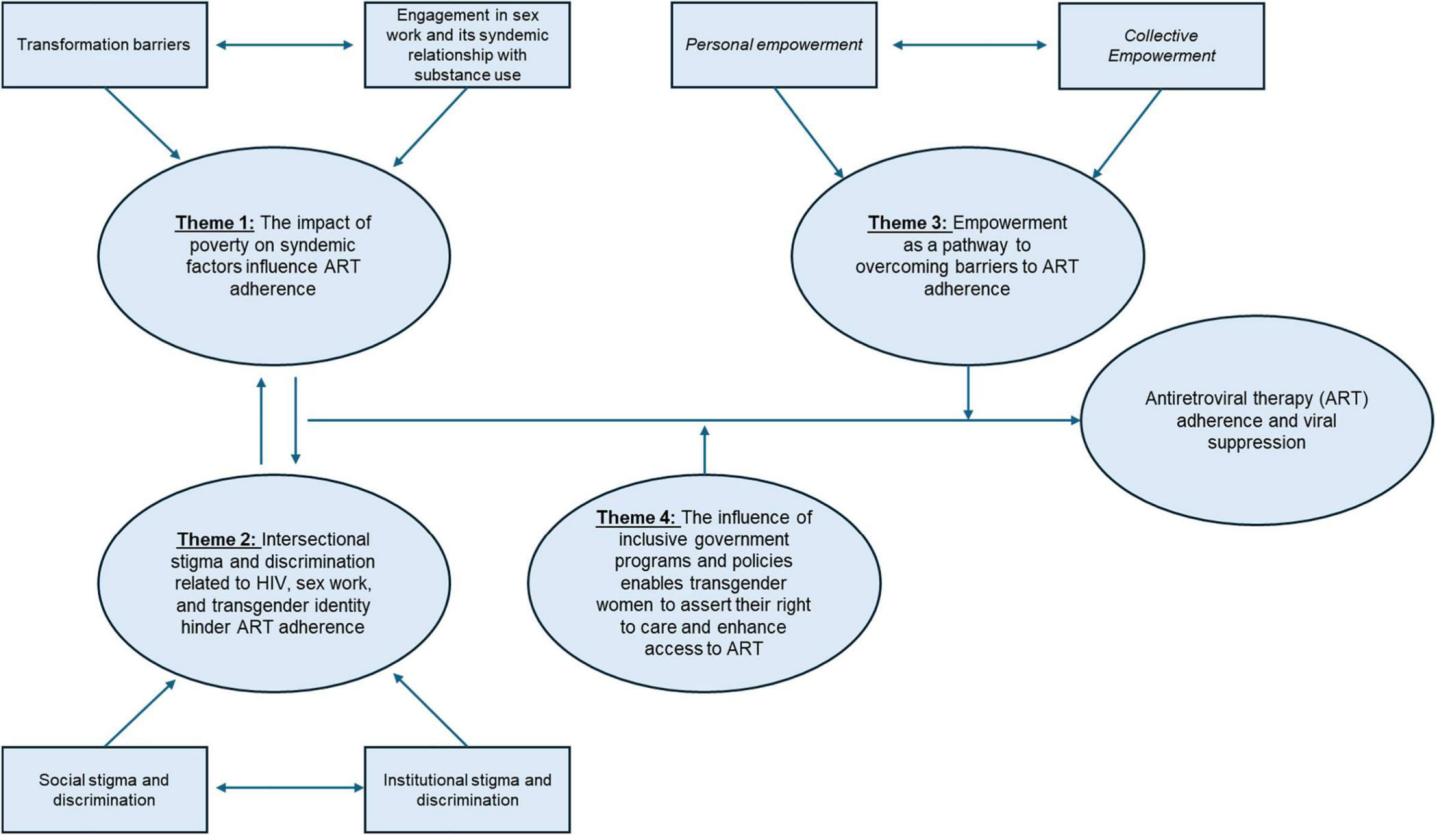
Visual diagram of themes and sub-themes impacting ART adherence and viral suppression.

Our research findings reveal four key themes derived from the in-depth interviews: (1) the impact of poverty on syndemic factors (i.e. mental health, substance use) influencing ART adherence, (2) intersectional stigma and discrimination related to HIV, sex work, and transgender identity hindering ART adherence, (3) empowerment as a means to overcome barriers to ART adherence, and (4) the influence of inclusive government programmes and policies enabling transgender women to assert their right to care and enhance access to ART. We organised the topic areas into barriers (Themes 1 & 2) and supportive factors (Themes 3 & 4). Each overarching theme contains sub-themes that cluster and interact with one another, influencing ART adherence and viral suppression (Figure 1).

#### The impact of poverty on syndemic factors influence ART adherence

##### Transportation barriers:

While the ‘test and treat’ policy provides free ART immediately after diagnosis, participants reported that structural barriers, such as transportation costs, impede their ability to fully benefit from this policy. One participant highlighted, ‘Sometimes it happens that I do not have enough money to go to the ART center to get the medicine. I live far from the ART center’ (Delhi, 012). Participants also recounted the psychological burden of choosing between personal health and other responsibilities such as family, food, and housing due to financial burden, exacerbating mental health conditions. One participant stated:

Yes, it has happened due to mental trauma. I was very mentally disturbed also. It was like, I know I have this problem, but I did not have money to go for the medicine. Then I had to look after my family … if I would take care of myself, I had to skip my family and vice versa (Delhi, 011).

The economic status of this population influences the decision to prioritise one’s health or one’s family. The participant highlights how poverty impacts access to ART, causing mental health distress. Other participants illuminate this synergistic relationship between poverty, mental health, and HIV. One participant stated, ‘Sometimes it happens that medicine is over, but I don’t have money. I don’t have money to pay travel expenses, then I get anxious about whom to approach for money (Mumbai, 002). The indirect cost of accessing essential HIV care due to financial constraints contributing to mental health conditions was seen throughout the interviews. These findings demonstrate that while the ‘test and treat’ policy facilitates ART initiation, the economic vulnerabilities of TGW compromise their ability to adhere to long-term treatment.

##### Engagement in sex work and its syndemic relationship with substance use:

To alleviate these financial constraints, many TGW living with HIV in our study engage in sex work or begging. However, despite these efforts, they still struggle with managing costs related to travel, nutrition, and housing, which hinders their access to care despite ART being free:

Because sex worker transgender women do not earn so much that they can spend on themselves. They have to pay the convenience cost, and they have to give money to their Gurus [i.e. the masters within the extensive kinship system in the hijra community] also. So they have problems managing traveling and nutrition and dietician costs (Delhi, 013).

The necessity of sex work as a form of income also creates a syndemic relationship between alcohol use, sex work, and its implication for ART adherence. Substance use, particularly alcohol consumption, emerged as a notable barrier for many participants. The fear of mixing alcohol with medication or missing doses due to intoxication was a recurring concern. A participant states, ‘I drank alcohol then it is said that I don’t take medicine’ (Delhi, 010). Participants also report the need for alcohol when engaging in sex work, which impacts their ART adherence as well. A participant described drinking as driven by the need to ‘get the courage to do sex work and to face anyone’. The participant continues, saying, ‘During that period, sometimes it also happened that I did not take the medicine on time’ (Mumbai, 001).

The interviews underscore how poverty, manifested in transportation barriers and the need for income through sex work and begging, contributes to syndemic barriers, including mental health conditions and substance use.

#### Intersectional stigma and discrimination related to HIV, sex work, and transgender identity hinder ART adherence

##### Social stigma and discrimination:

Throughout the interviews, participants described strategies to navigate the stigma and discrimination associated with their marginalised statuses. Social stigma and discrimination pose significant challenges for many participants as they grapple with the intersection of HIV, sex work, and transgender identity. For example, when asked about her primary concern living with HIV, one participant expressed, ‘hiding my status from community people’ (Delhi, 015). The societal perception of HIV and transgender identity fosters a pervasive fear among participants, compelling them to conceal their status to avoid additional discrimination. For example, a participant shared an experience of being labelled as having a ‘dirty disease’ because, in the minds of others, ‘trans means sex work only and nothing else’ (Delhi, 006). These intersecting social positions often leads to individuals resorting to taking their medication covertly and, in some instances, missing prescribed doses. A participant describes how she and other TGW living with HIV are treated within the community:

Actually, in the transgender community, if anyone is HIV positive, then they look at her with hate … so [people living with HIV] do not open up, and they are not ready to take medicine because they have to live with [other transgender women] and it becomes a problem to keep medicine there. They gossip about which tablet you are taking. So [people living with HIV] are scared (Mumbai, 010).

The participant highlights how stigma and discrimination within the transgender community instil fear based on their identity, leading many to conceal their medication use, but it also impacts their ART adherence.

Additionally, many participants recount being rejected upon disclosing their status by family, peers, and friends, including being instructed to sleep and eat separately. One participant stated:

At that time, I told my family [that I was living with HIV], and after telling them … they said you have visited dirty places and brought this disease. My parents tore up my green card for HIV and threw it away. After that, I had to struggle a lot after stopping medicine (Delhi, 006).

In this account, the family not only rejected the participant but also destroyed their Green Book, a vital document for receiving HIV treatment in India. This act of destroying medical records and hindering access to life-saving services constitutes a form of violence and systemic abuse, effectively disabling access to ART.

##### Institutional stigma and discrimination:

Despite the ‘test and treat policy focus on improving ART linkage, participants frequently reported facing stigma within the healthcare setting, limiting its effectiveness. Participants faced institutional discrimination based on their transgender identity and HIV status, experiencing discrimination from doctors, staff, and fellow patients at the health facility. A participant shared her initial encounter at an ART centre:

My experience at the ART center has not been good. From the very beginning, I was facing stigma and discrimination … They have a mentality that trans people are involved in sex work only, and they have to get this disease for sure … people see and judge that it has happened to me because of sex work. It is not necessary that all trans women are doing sex work. So, counselors and doctors tell us to stay away. They do not allow us to come near, and they do not listen to us regarding our problem (Delhi, 008).

The societal perception of TGW as sex workers permeates the health care setting and impacts the treatment TGW receive in their HIV care. Another participant stated,

[Health care workers] there misbehaved, pointing out that I engage in sex work and have HIV. They used to say very dirty things and used to abuse also … My starting experience was not good … nobody was supporting (Delhi, 011).

Many participants describe this mistreatment due to their intersecting identities, highlighting how it undermines their medication adherence while also revealing how stigma in spaces intended for care results in neglect and a lack of adequate support. One participant said, ‘They discriminate and treat us differently. Very often, when I go to collect my medicine, they tell me to wait and prioritise others … trans people feel uncomfortable taking medication in the presence of non-community individuals’ (Delhi, 012). The pervasive institutional stigma and discrimination faced by participants, rooted in societal perceptions of transgender women as sex workers, not only compromise the quality of healthcare interactions but also emerge as barriers to sustained ART adherence and ongoing HIV care. Additionally, it highlights the ways stigma at ART centres undermines the policy’s potential to enhance HIV care access.

#### Empowerment as a pathway to overcoming barriers to ART adherence

Empowerment among TGW living with HIV stems from the interplay of personal determination and collective efforts. Empowerment provides the foundation for overcoming barriers to ART adherence by addressing both internal resilience and external support systems, demonstrating its critical role in improving ART adherence among TGW living with HIV.

##### Personal empowerment:

Many participants faced social and familial rejection due to HIV and transgender stigma; however, they understood the importance of taking their medication as prescribed in the face of these obstacles. A participant identifying as Kinnar – an identity similar to Hijra – highlighted the unique struggles associated with their societal position, stating:

If I were a boy, I would have earned for [my parents]; if I were a girl, I would have gone to my in-law’s place, but today, I am a Kinnar. If I did not do anything for them, I would die useless. So, I decided to live with my confidence. I had confidence, and I had support … You must live your life; if you have to make your way, take medicine on time. If we do not take the tablet on time, how will we survive (Mumbai, 002).

The participant illustrates how belonging to a supportive community and embracing her identity as Kinnar empowered her to reclaim agency over her life and prioritise her health. While economic instability often left participants feeling marginalised, her sense of personal empowerment allowed her to reclaim agency over her life through HIV care, emphasising the critical importance of adhering to medication to sustain health and survival. Despite the challenges associated with her social position as a Kinnar, she found a sense of stability and purpose in maintaining her health.

Similarly, another participant described the importance of taking their medication: ‘Transwomen should become capable of taking medicine by themselves because how long will the NGO people be there. If we don’t become self-dependent, we will be careless, and we can’t do anything then’ (Delhi, 012). The participant underscores the importance of self-determination, recognising that external support from NGOs may not always be available. This sentiment reflects a deeper connection to their community, where resilience and mutual support inspire individuals to remain committed to their medication regimen despite systemic barriers.

##### Collective Empowerment:

While personal empowerment reflects TGW’s internal resilience, collective empowerment highlights the external structures of support that amplify their ability to overcome systemic barriers. TGW living with HIV discussed their interconnected social support networks, which collectively facilitate ART adherence. These networks included family and peers, community-based organisations, and health providers. All of which contributed to a sense of collective empowerment for the participants. Each of these networks contributed uniquely to participants’ empowerment, creating a multifaceted system of support.

First, for some participants, their family was not a source of stigma and rejection but rather a source of support in helping them with ART adherence. A participant describes going into the city from her village and telling relatives that she is going for her medicine: ‘My relatives are educated … They said that you keep taking medicine till you are alive and take care of your diet, etc. They also did not discriminate’ (Delhi, 008). In addition to family, kinship systems and peers were crucial support systems for TGW, helping to address both logistical and emotional barriers to ART adherence. A participant shared, ‘Our guru is very nice, and she knows about my illness. When my medicine runs out, I tell my guru one day before, and I will take leave tomorrow because I have to go to get medicines’ (Delhi, 005). The participant highlights how the kinship systems within the hijra community foster collective accountability and provide tangible assistance, such as ensuring timely access to ART. These kinship networks exemplify informal collective support, where shared experiences and mutual accountability help overcome barriers to care.

Second, relationships with community-based organisations were also instrumental in adhering to ART among the participants. They discussed getting healthcare workers to refer them to CBOs for care, helping them get medication when they ran out, and providing emotional and financial support, stating, ‘[Healthcare worker] has done a great favour to me by referring me [to CBO]. I want to thank him for bringing me here, and now I get medicine here … People here are very good and talk to me very nicely. Whenever I come here, I get the support’ (Mumbai, 007). The participant illustrates how CBOs create safe and inclusive spaces while also providing critical resources to overcome structural barriers to adherence.

Finally, while many participants described facing discrimination at healthcare facilities, others described positive and empowering relationships with healthcare providers, especially doctors, as key to improving adherence. One participant describes, ‘I consider doctor as God … Only a doctor understands our body well and counsels us to take this or that (Delhi, 003). This type of relationship is built and based on shared understanding and mutual respect. A participant recounts her experience,

I know about myself, and since I talk to the doctor very lovingly, the doctor also will talk to me nicely. If we clap and misbehave, the doctor will also say something to us for sure … I go there and get the medicine easily (Delhi, 013).

#### The influence of inclusive government programs and policies enables transgender women to assert their right to care and enhance access to ART

Legal recognition of the third gender status, as described by a participant, allows them to leverage their legal recognition to demand HIV care, ‘Now hijra community has become smart, and they know how to get treatment. Now, we move ahead, clap, and get fast treatment. We do not step back and will not be afraid ‘(Mumbai, 010). The knowledge and awareness of policies designed to protect this community empower participants to be able to demand quality HIV care, leading to a transformative impact on previously discriminatory practices. Participants highlight changes to ART clinics since TGW began advocating for themselves and challenging discriminatory practices. A participant notes:

So many changes have come because our community has done advocacy there, and they educated them about us that if you support us then only when we will take medicine, otherwise we won’t … Earlier, there was discrimination. They used to say, stand that side, and we will allow you later. But now their behavior has changed (Mumbai, 013).

Notably, there was confusion about government schemes for supporting TGW living with HIV. A participant describes the importance of informing people of these government schemes, ‘First of all, we should tell them that the government is providing help to them with great difficulty … The government supports positive transgender people or anyone positive in the LGBT community’. (Mumbai, 09). However, a participant highlighted the need to inform others of these schemes and how structural barriers prevent TGW from accessing the government schemes, as described:

Many TGW have been positive for a long time and are on medication. They are still unaware that the government is giving 2500 rupees to positive people. Then gimmicks are sitting in the ART center saying your address is wrong, your bank details are wrong, and this and that (Delhi, 009).

Through the interviews, participants emphasised how advocacy and policy interventions are crucial to support their adherence to ART; however, many participants noted a need to help improve the community’s understanding and ability to access these schemes due to structural issues such as bank accounts and stable housing.

## Discussion

This study contributes to the limited literature on barriers and supportive factors to ART adherence among TGW living with HIV in India (Beattie et al., 2012; Chakrapani et al., 2023). We identified two barriers and two supportive factors impacting ART adherence: poverty and intersectional stigma as barriers and empowerment and inclusive government policies as facilitators. These themes underscore how intersecting social positions – such as gender identity, sex worker status, and HIV status – interact with broader systems of oppression (e.g. capitalism) to influence ART adherence (Bowleg, 2021; Crenshaw, 1991).

Our findings illuminate how poverty influences ART adherence through structural and individual-level barriers and how these barriers are inextricably linked and influence each other. Although the ‘test and treat’ policy was designed to improve ART initiation by providing free and immediate access to ART, our data suggest that structural barriers, such as transportation cost or economic vulnerabilities, continue to impede adherence among TGW living with HIV. Poverty and economic vulnerability create structural barriers to ART adherence, including challenges like transportation costs to ART clinics. These findings align with similar studies conducted in India and globally (Chakraborty et al., 2020; Colocci et al., 2021; Eshun-Wilson et al., 2019; Isabel Steinert et al., 2022; Joglekar et al., 2011; Lankowski et al., 2014; Modi et al., 2022; Nyamathi et al., 2011; Shastri et al., 2013). Additionally, poverty impacts adherence to ART through individual-level barriers. Our study highlights how participants grapple with mental health conditions linked to economic scarcity. They face the difficult choice between providing for themselves (i.e. prioritising their HIV costs) or their families. Moreover, economic hardship compels many TGW living with HIV to engage in sex work, contributing to alcohol use as a coping mechanism.

This research expands on the literature concerning the impact of poverty on ART adherence, emphasising the connection between syndemic conditions, such as mental health conditions and alcohol use, and poverty among TGW. Our findings highlight a disconnect between the policy’s goal and the reality of sustained ART adherence. Comprehensive interventions are needed to address these multilevel factors from benefiting from the ‘test and treat’ policy that will not only show short-term but long-term improvements in HIV care. For example, diversifying ART delivery approaches such as offering ART pick-up services from community-based sites, gender sensitisation training for health providers and staff, extending government ART operational hours, enabling multi-month ART dispensing, and providing nutrition support could enhance access to ART among TGW living with HIV in India. These improved strategies have the potential to improve mental health by reducing the psychosocial stress related to economic vulnerability and the need to select between paying for personal versus family priorities. These approaches proved effective for key populations during the COVID-19 pandemic (Hung et al., 2022; National AIDS Control Organization, 2020; Pollard et al., 2021; Samudyatha et al., 2022). Thus, the continuation of these alternative approaches is needed for a comprehensive and flexible healthcare delivery model for TGW living with HIV.

Notably, the Government of India Ministry of Social Justice & Empowerment has implemented policies to address economic disparities among TGW through the comprehensive scheme called Support for Marginalised Individuals for Livelihood and Enterprise (SMILE)(Government of India Ministry of Social Justice & Empowerment, 2022). This initiative aims to enhance the economic well-being of transgender individuals by offering scholarships for transgender students, skill development programmes, medical facilities, counseling services, and economic linkages to employment outside of sex work and begging. It is crucial to recognise that while these initiatives exist, further research is necessary to monitor and evaluate how policies like SMILE contribute to improving economic status and ART adherence among TGW living with HIV in India. Also, TGW living with HIV must be aware of these programmes and policies so that they can advocate for themselves, as we see in the data.

Additionally, our study also shows that TGW living with HIV experience intersecting stigmas that impact ART adherence related to their transgender identity, HIV status, and sex work, similar to limited studies within and outside of India (Chakrapani et al., 2023; Lacombe-Duncan et al., 2020; Logie et al., 2019). Similar to Chakrapani et al., we found intersecting stigma within and outside of the TGW community related to TGW living with HIV, resulting in social isolation, unstable housing, and familial rejection and often leading to fear of disclosure (Chakrapani et al., 2023). Our studies highlight the need for interventions in India that combat intersectional stigma to improve HIV care. While the ‘test and treat’ policy addresses ART access, it does not explicitly target the intersectional stigmas that TGW face, particularly within healthcare settings, where stigma can undermine the policy’s intent to facilitate equitable care. Interventions focused on reducing stigma through community-level advocacy and education, as suggested by Andersson et al. (2020) (Andersson et al., 2020).

Despite various barriers, participants highlighted factors that improve ART adherence, emphasising personal and collective empowerment. Our study builds upon the groundwork laid by Blanchard et al. (2013) among female sex workers in South India, echoing the significance of empowerment, denoted as three constructs: the power within (such as self-esteem and confidence), the power with (indicative of collective identity and solidarity), and the power over (access to social entitlements) in managing HIV-related risks (Blanchard et al., 2013). This framework is particularly relevant to the experiences of TGW in our study, who described personal empowerment (‘power within’) as critical for adherence, such as developing self-confidence to prioritise health. Collective empowerment (‘power with’) was evident in participants’ reliance on community-based organisations and peer networks to access ART. Additionally, participants leveraged legal recognition to exercise ‘power over’ healthcare systems, demanding equitable care and advocating for policy changes. Our findings advocate for interventions aligned with Blanchard’s Integrated Empowerment Framework to enhance community mobilisation and HIV care among TGW living with HIV. Our data reveal that participants are aware of their power to adhere to ART. This awareness suggests that empowerment interventions rooted solely in individual resilience or self-efficacy may not achieve sustained impact. Instead, community mobilisation remains central to fostering empowerment, as it builds the collective solidarity and shared resources that enable TGW living with HIV to navigate structural barriers.

### Limitations

Although this study offers valuable insights, it is not without limitations. The research focused specifically on TGW living with HIV in Mumbai and New Delhi, India. These cities have mature HIV interventions and robust community-based groups. Consequently, the findings may not fully capture the experiences and lived realities of transgender women in non-urban settings with less established HIV care and support programmes and weaker community presence. Additionally, the study was conducted in Hindi, Marathi, and English, potentially limiting the findings to TGW living with HIV who speak other languages.

## Conclusion

In conclusion, our study provides valuable insights into the challenges faced by TGW living with HIV in Mumbai and New Delhi, particularly about their adherence to ART following the implementation of the ‘test and treat’ policy. A holistic approach is essential to addressing the interconnected challenges faced by TGW living with HIV in India. Through this study, we aim to contribute to informed interventions, policies, and healthcare practices.

Our findings underscore the importance of empowering TGW through personal and collective empowerment, addressing intersectional stigma, and evaluating policies aimed at reducing poverty. Targeted strategies, such as economic empowerment programmes and mental health support initiatives, are essential to improving ART adherence among TGW. Addressing stigma may involve implementing community education and advocacy initiatives to foster inclusion and reduce discrimination. Furthermore, evaluating policies to alleviate poverty requires thoroughly examining programmes like SMILE. By tackling these multifaceted challenges, this study contributes to the broader goal of equitable and inclusive HIV care, laying the groundwork for future research and policy innovation.

## Supplementary Material

Supp 1

Supplemental data for this article can be accessed online at https://doi.org/10.1080/17441692.2025.2473446.

## Figures and Tables

**Table 1.** Sociodemographic characteristics of transgender women living with HIV from in-depth interviews in Mumbai and New Delhi, India (*N* = 30).

	Overall N (%)
**City = New Delhi (%)**	15 (50%)
**Age**	
18–24 years old	3 (10%)
25–34 years old	11 (37%)
35–44 years old	8 (27%)
45–54 years old	7 (23%)
55–64 years old	1 (3%)
**Gender Identity**	
Hijra	11 (37%)
Transgender Women	19 (63%)
**Education**	
Literate but no formal schooling	6 (20%)
SCC (Secondary school Certificate) and HSC Higher Secondary Certificate	18 (60%)
Some college, but no graduate	1 (3%)
Graduate/Postgraduate (General)	4 (13%)
Graduate/Postgraduate (Professional)	1 (3%)
**Religion**	
Hindu	27 (90%)
Muslim	3 (10%)
**Caste status**	
Scheduled caste (SC)	9 (30%)
Scheduled Tribe (ST)	1 (3%)
Other backward class (OBC)	5 (17%)
General Category	14 (47%)
Do not know	1 (3%)
**Visual conformity with affirmed gender or ‘passing’.**	
Always	9 (30%)
Most of the time	4 (13%)
Sometimes	8 (27%)
Rarely	8 (27%)
Never	1 (3%)
**Perceived skin color**	
Very fair	1 (3%)
Fair	7 (23%)
Medium/Dusky	17 (57%)
Dark	5 (17%)
**Monthly income**	
Less than 5000 INR	3 (10%)
5,000–10,000 INR	9 (30%)
10,000–15,000 INR	9 (30%)
15,000–20,000 INR	7 (23%)
More than 20,000 INR	2 (7%)
**History of engagement in sex work = Yes**	30 (100%)
**Sex work in the past 3 months**	
No	7 (23%)
Yes	22 (73%)
Prefer not to answer	1 (3%)
**Currently on ART = Yes**	30 (100%)
**ART adherence in the past month**	
Fair	4 (13%)
Good	11 (37%)
Very good	4 (13%)
Excellent	11 (37)
**ART adherence in the past month (mean(SD))**	0.91 (0.10)
**ART adherence above 90% in the past month**	24 (80%)
**ART adherence = 100% in the past month**	12 (40%)
