# Supplementary material for: From policy to practice: syndemic and intersectional challenges to ART adherence for transgender women under India’s post-test and treat policy
Source: Glob Public Health. Author manuscript; Available in PMC 2025 Dec 1. (PMC12266041; doi:10.1080/17441692.2025.2473446)
Supplement: Supp 1 [file NIHMS2065822-supplement-Supp_1.docx]

1. **INTERSECTING SOCIAL POSITIONS AND INEQUITIES**
   1. **TG + HIV + Perceived skin color**
   2. **TG + HIV + Religion**
   3. **TG + HIV + Education**
   4. **TG + HIV + Caste**
   5. **TG + HIV + Visual conformity**
   6. **TG + HIV + Sex work**
2. **SYNDEMIC BARRIERS TO ART ADHERENCE**
   1. **General Health**
      1. **Non-communicable diseases**
      2. **STI**
      3. **Physical Health**
      4. **Environmental Health**
   2. **Individual level factors**
      1. **Internalized transphobia**
      2. **Mental health**
      3. **Substance & alcohol use (licit and illicit)**
      4. **Suicide ideation**
   3. **Interpersonal level**
      1. **HIV-related stigma/discrimination**
         1. **within the TG community**
         2. **among family & friends**
      2. **Violence (i.e., IPV, community, police, familial)**
         1. **IPV/Client**
         2. **Community**
         3. **Police/Authority**
         4. **Familial**
      3. **Stigma/discrimination**
         1. **Gender identity**
         2. **Caste**
         3. **Visual conformity**
         4. **Substance use**
   4. **Structural level**
      1. **Discrimination at the health facility**
         1. **Gender**
         2. **HIV**
      2. **Housing**
      3. **Poverty**
      4. **Cost**
         1. **Transportation costs**
         2. **Non-transportation costs**
      5. **Disrespect/Treated poorly**
      6. **Maintenance/Facility**
      7. **ART not available at health facilities**
   5. **Intersecting and clustering of syndemic factors**
3. **OTHER BARRIERS TO ART ADHERENCE**
   1. **Storing medication**
   2. **Taking medication in a private setting (i.e., away from family or/and friends)**
   3. **Forgetting**
   4. **Work (i.e., sex work, dancing, begging, NGO)**
      1. **Sex work**
      2. **Dancing**
      3. **Begging**
      4. **NGO**
   5. **Living with Family**
   6. **Side effects/risks/toxicity**
   7. **Travel**
4. **FACILITATORS TO ART ADHERENCE**
   1. **Social support**
      1. **Family**
      2. **Peer**
      3. **Organization**
   2. **Resilience**
   3. **Strategies for medication adherence**
   4. **Public schemes to support PLWHIV**
5. **COVID**
6. **HIV TREATMENT MISINFORMATION/RUMORS**
7. **FUTURE INTERVENTIONS**
   1. **Use of technology**
   2. **Leadership model**
      1. **Peer-led**
      2. **Government-led**
      3. **NGO/CBO-led**
   3. **Format**
      1. **Individual**
      2. **Group**
      3. **Combination**
   4. **Different ART modalities**
   5. **Counseling or behavioral interventions**
   6. **TG specific program**
   7. **Health communication**
8. **HIV TREATMENT SERVICES**
9. **STATUS DISCLOSURE**

1. **GREAT QUOTES**
2. **TOPICS FOR DISCUSSION WITH THE TEAM**

**DETAILED CODEBOOK**

| **NAME** | **DESCRIPTION** | **NOTES/QUOTE EXAMPLE** |
| --- | --- | --- |
| **1. INTERSECTING SOCIAL POSITIONS AND INEQUITIES** | Beliefs, experiences, and expectations related to one’s own social positions and inequities in relationship to HIV care | **This can include ART adherence or experiences receiving HIV treatment.**  INCLUDES PERCEPTIONS BASED ON EXPERIENCES OF FRIENDS AND ACQUAINTANCES |
| 1.1 TG + HIV + Perceived skin color | Participants’ experiences and beliefs of being TG, living with HIV, and colorism and its impact on HIV care |  |
| 1.2 TG + HIV + Religion | Participants’ experiences and beliefs of being TG, living with HIV, and belonging to a specific religion and its impact on HIV care |  |
| 1.3 TG + HIV + Education | Participants’ experiences and beliefs of being TG, living with HIV, and education status and its impact on HIV care |  |
| 1.4 TG + HIV + Caste | Participants’ experiences and beliefs of being TG, living with HIV, and belonging to a specific caste and its impact on HIV care |  |
| 1.5 TG + HIV + Visual conformity | Participants’ experiences and beliefs of being TG, living with HIV, and ability to visually conform or not and its impact on HIV care |  |
| 1.6 TG + HIV + Sex work | Participants’ experiences and beliefs of being TG, living with HIV, and engaged in sex work and its impact on HIV care |  |
| **2. SYNDEMIC BARRIERS TO ART ADHERENCE** | Any discussion of multilevel syndemic barriers to ART adherence. We are also interested in how these barriers intersect to worsen health outcomes/ART adherence. |  |
| 2.1 General Health | Discussions about their general health status and health concerns that impact them and other transgender women in their community. |  |
| 2.1.1 Non-communicable diseases | Discussion of non-communicable diseases (e.g., cancers, cardiovascular disease, diabetes, and chronic lung illnesses) |  |
| 2.1.2 STI | Discussion of STI or experience with STI testing |  |
| 2.1.3 Physical Health | Discussion of physical health. Physical health involves many aspects of life, such as: sleeping well, eating well, being physically active, having good hygiene, and getting enough relaxation. | SHOULD NOT BE USED FOR IMPLICIT TRUST IN PROVIDERS/ CLINICS. |
| 2.1.4 Environmental Health | Discussion of environmental health. Environmental health involves how air quality, noise pollution, healthy housing, food safety, and waste management impact one's health. |  |
| 2.2 Individual level factors | Discussion of how individual-level factors (i.e., mental health, substance use, suicide ideation) impact ART adherence. |  |
| 2.2.1 Internalized transphobia | Any experience with internalized transphobia. Internalized transphobia is discomfort with transgender identity due to internalizing society's normative gender expectations. |  |
| 2.2.2 Mental Health | Any experience with mental health conditions (diagnosed by a provider or not). |  |
| 2.2.3 Substance & alcohol use (licit and illicit) | Any discussion of alcohol or other substances (licit and illicit), either lived experiences, hypothetical ones, attitudes, beliefs, etc. Includes use of alcohol and substances before, during, or after sex |  |
| 2.2.4 Suicide ideation | Any discussion or experiences with suicidal ideation, or suicidal thoughts, which is the thought process of having ideas or ruminations about the possibility of ending one's own life |  |
| 2.3 Interpersonal level | Any discussion of interpersonal (violence, abuse, trauma) and societal (stigma) factors that impact ART adherence. Peers, community, society, and family can attribute interpersonal factors. These can be the experience of the participant or community. |  |
| 2.3.1 HIV-related stigma/discrimination | Any discussion of experiences, beliefs, or attitudes of HIV-related stigma/discrimination and its impact on HIV care. WE ARE NOT INCLUDING HIV-RELATED STIGMA WITHIN THE HEALTHCARE SETTING |  |
| 2.3.1.1 within the TG community | Any discussion of experiences, beliefs, or attitudes of HIV-related stigma/discrimination within the TG community and how it impacts HIV care/ART adherence (more specifically) |  |
| 2.3.1.2 within the TG community | Any discussion of experiences, beliefs, or attitudes of HIV-related stigma/discrimination among family & friends and how it impacts HIV care/ART adherence |  |
| 2.3.2 Violence | Experiences of intimate partnered violence, community violence, violence by the police, or within the family and how that impacts ART adherence |  |
| 2.3.2.1 IPV/Client | Experience of intimate partnered violence or violence from client |  |
| 2.3.2.2 Community | Experience of violence from the community (i.e., other H/TG) or peers |  |
| 2.3.2.3 Police/Authority | Experience of violence from the police or authority figure |  |
| 2.3.2.4 Familial | Experience of violence from family members |  |
| 2.3.2 Transgender stigma/discrimination | Discussion of transgender stigma/discrimination, which includes discrete events of discrimination and victimization (experienced or enacted stigma) and/or feelings of devaluation and expectations of hostility (felt, perceived, or anticipated stigma) |  |
| 2.3.3.1 Gender identity | Experience of transgender stigma based on gender identity |  |
| 2.3.3.2 Caste | Experience of transgender stigma based on caste |  |
| 2.3.3.3 Visual conformity | Experience of transgender stigma based on visual conformity |  |
| 2.3.3.4 Substance use | Experience of transgender stigma based on substance use |  |
| 2.4 Structural level | Discussion of structural violence that results in castism, colorism, substandard/unstable housing, unmet gender-affirming care, and poverty, leading to barriers preventing TG communities from accessing HIV care. |  |
| 2.4.1 Discrimination | Experiences of discrimination or unmet gender-affirming care at health facility impacting access to ART or ART adherence |  |
| 2.4.1.1 Gender | Experience of discrimination based on gender at health facilities |  |
| 2.4.1.2 HIV | Experience of discrimination based on HIV at health facilities |  |
| 2.4.2 Housing | Discussion of substandard/unstable housing and its impact on HIV care/ART adherence |  |
| 2.4.3 Poverty | Discussion of poverty and its impact on HIV care/ART adherence |  |
| 2.4.4 Cost | Discussion of the cost of accessing ART medication |  |
| 2.4.4.1 Transportation costs | Experience of spending money for transportation to ART clinic or facility that they pick up medication |  |
| 2.4.4.1 Other Transportation costs | Experience of spending money to access ART. For example: take it takes to get there or convenience of location |  |
| 2.4.5 Disrespect/Treated poorly | Discussion of being treated poorly or disrespected at a health facility. |  |
| 2.4.6 Maintenance/Facility | Discussion of the facility or maintenance of the health facility being poor and how that contributes to ART |  |
| 2.4.7 ART not available at health facilities | Discussion of health facilities having shortage of medication |  |
| 2.5 Intersecting and clustering of syndemic factors | Any discussion of intersecting and clustering syndemic factors.  **REMINDER:** Syndemic theory was developed by Merrill Singer; it postulates that adverse social structures (e.g., poverty, discrimination), in conjunction with multiple co-occurring psychosocial factors such as mental health conditions and substance use, synergistically exacerbate poor health. |  |
| **3. OTHER BARRIERS TO ART ADHERENCE** | Topics related to participants’ barriers to ART adherence that aren’t psychosocial and structural but have to do with the administration of the medication and side effects |  |
| 3.1 Storing medication | Discussion of storing medication and its impact on ART adherence |  |
| 3.2 Taking medication | Any discussion of taking medication privately, as in away from family, guru, peers, or/and friends. We should also include attitudes toward taking medication. |  |
| 3.3 Forgetting | Any discussion of forgetting to take medication |  |
| 3.4 Work | Any discussion of work (i.e., sex work, begging, dancing, NGO work) getting in the way of work |  |
| 3.5 Living with family or in Dera | Any discussion of the difficulty of taking medication because the participant lives with family or a guru |  |
| 3.6 Side effects/risks/toxicity | Discussions or concerns about perceived side effects/safety/toxicity of ART medication |  |
| 3.7 Travel | Discussion of being out of town and unable to get ART.  [NOT ABOUT TRAVELING TO ART CLINIC] |  |
| **4. FACILITATORS OF ART ADHERENCE** | **Any discussion of facilitators that help TG or the TG community adhere to ART. They can consist of social support, resilience, technology, or public schemes that support PLHIV.** |  |
| 4.1 Social Support | Discussion of family/NGO/peer support in adhering to ART medication as prescribed. |  |
| 4.2 Resilience | Discussion of resilience is often defined as related to an individual’s “positive adaptation within the context of significant adversity” and how that empowers them to adhere to ART. |  |
| 4.3 Strategies for medication adherence | Participants discuss **concrete** strategies to maintain adherence to ART, such as setting an alarm. |  |
| 4.4 Public schemes to support PLHIV | Participants discuss schemes/programs the government provides (i.e., food vouchers, travel reimbursements) that help them adhere to ART. |  |
| **5. COVID** | Any mentions of the coronavirus or changes in sexual or medication-taking behaviors attributed to COVID-19, social distancing, etc. It also includes changes in healthcare access due to the coronavirus pandemic. |  |
| **6. HIV TREATMENT MISINFORMATION/RUMORS** | **Any** discussions of misinformation or rumors heard regarding HIV care/treatment/ART adherence. |  |
| **7. FUTURE INTERVENTIONS** | Any discussion of future interventions that would be useful to improve ART adherence for themselves or the community. |  |
| 7.1 Use of technology | Any discussion of the use of technology to improve ART adherence, including setting alarms, WhatsApp groups, etc. |  |
| 7.2 Peer-led or government | Discussion of whom they would like to be able to run the program/intervention |  |
| 7.3 Format | Discussion of the intervention/program format, including but not limited to individually, group, or a combination of both. |  |
| 7.4 More TG-specific Interventions/NGOs | Discussion of TG-specific interventions/NGOs that they would consider interventions to mirror and why. |  |
| **8. GREAT QUOTES** | Any standout quotes that would be useful for manuscripts or grants; quotes that raise new or interesting topics that could warrant additional analysis or team discussion. | It should be double-coded with other codes whenever possible.  Must be annotated with a memo re: significance and relationship to possible themes, key findings, or emergent topics. |
| **9. TOPICS FOR DISCUSSION WITH THE TEAM** | Use this code to flag topics to discuss in coding meetings.  Add a memo specifying the topic you want to discuss with the team. |  |
